# Still Organizing

**Published:** 1887-10

**Authors:** 


STILL ORGANIZING.—The next meeting of the Terrell Medical Society will be held at Forney, when, it is expected, the profession of that city and vicinity will come into the fold. Success.

